# Novel insights into the interaction of UBA5 with UFM1 via a UFM1-interacting sequence

**DOI:** 10.1038/s41598-017-00610-0

**Published:** 2017-03-30

**Authors:** Prasanth Padala, Walaa Oweis, Bayan Mashahreh, Nadine Soudah, Einav Cohen-Kfir, Emily A. Todd, Christopher E. Berndsen, Reuven Wiener

**Affiliations:** 1 Department of Biochemistry and Molecular Biology, the Institute for Medical Research Israel-Canada, Hebrew University-Hadassah Medical School, Jerusalem, 91120 Israel; 2 Department of Chemistry and Biochemistry, James Madison University, Harrisonburg, Virginia VA 22807 USA

## Abstract

The modification of proteins by ubiquitin-fold modifier 1 (UFM1) is implicated in many human diseases. Prior to conjugation, UFM1 undergoes activation by its cognate activating enzyme, UBA5. UBA5 is a non-canonical E1 activating enzyme that possesses an adenylation domain but lacks a distinct cysteine domain. Binding of UBA5 to UFM1 is mediated via an amino acid sequence, known as the UFM1-interacting sequence (UIS), located outside the adenylation domain that is required for UFM1 activation. However, the precise boundaries of the UIS are yet not clear and are still under debate. Here we revisit the interaction of UFM1 with UBA5 by determining the crystal structure of UFM1 fused to 13 amino acids of human UBA5. Using binding and activity assays, we found that His 336 of UBA5, previously not reported to be part of the UIS, occupies a negatively charged pocket on UFM1’s surface. This His is involved in UFM1 binding and if mutated perturbs activation of UFM1. Surprisingly, we also found that the interaction between two UFM1 molecules mimics how the UIS binds UFM1. Specifically, UFM1 His 70 resembles UBA5 His336 and enters a negatively charged pocked on the other UFM1 molecule. Our results refine our understanding of UFM1-UBA5 binding.

## Introduction

Modification of proteins by the addition of ubiquitin or ubiquitin-like proteins (UBLs) is crucial for proper functioning of cells. This process plays a key role in wide variety of cellular functions, such as cell division, antiviral response, DNA damage response, immune response and protein degradation^1–3^. Insufficiencies in the functioning of these modifications lead to many diseases ranging from cancer to neurodegenerative diseases^4, 5^. The addition of UBLs to target proteins, similar to ubiquitin, is carried out by a series of reactions. These reactions are catalyzed by three groups of enzymes, namely E1 (an activating enzyme), E2 (a conjugating enzyme) and E3 (a ligase enzyme). The activating enzyme E1 catalyzes the adenylation of the UBL C-terminus leading to thioester bond formation with the E1 active site cysteine. The charged E1 then transfers the UBL to the active site cysteine of an E2 enzyme in a transthioesterification reaction. Finally, with the assistance of an E3, the E2 transfers the UBL typically to a lysine residue on a target protein^6, 7^.

To date, eight E1 enzymes are known, each with specificity toward a particular UBL or ubiquitin. These enzymes are classified into two groups: canonical and non-canonical activating enzymes^8^. The major difference between the two classes is that the canonical E1 enzymes contain two separate domains known as the adenylation and the catalytic Cys domains while the non-canonical E1 enzymes possess only the adenylation domain, which also contains the catalytic cysteine. The non-canonical E1 group comprises three enzymes ATG7, UBA4 and UBA5 that are the cognate activating enzymes for the UBLs ATG8/12, URM1, and UFM1, respectively.

Ubiquitin fold modifier 1 (UFM1) exists in plants and animals but is absent in fungi^9^. Recent studies have revealed that UFM1 plays a role in many cellular processes including fatty acid metabolism, ER stress and erythroid development^10–15^. It was also shown that UFM1 is involved in human diseases including cancer, diabetes, schizophrenia, and ischemic heart diseases^11, 15–17^.

UFM1 is activated by UBA5, which in humans has two isoforms; one starts at the beginning of the adenylation domain (amino acid 57) and one has an extension of 56 amino acids N-terminal to the adenylation domain^18, 19^. In the activation process UBA5 adenylates UFM1 C-terminal Gly and then forms a thioester bond with the UFM1 C-terminus via its active site Cys250. Following activation, UFM1 is transferred from UBA5 to the E2, UFC1, which is to date the single known E2 of UFM1. Then, together with the E3, UFL1, UFM1 is transferred to its target protein^20–22^.

As a non-canonical E1, the active site cysteine (Cys250) of UBA5 is within the adenylation domain. This suggests that this domain, which ends at amino acid 329, satisfies UFM1 activation^23^. However, Xie has demonstrated that an extension of 34 amino acids C-terminal to the adenylation domain is required for UFM1 activation^24^. Furthermore, based on the secondary structure prediction, Xie proposed that this extension adopts a helical structure^24^. Last year, Habisov *et al*. used a UBA5 peptide array to further characterize the residues required for UFM1 binding and suggested that UBA5 binds UFM1 via an amino acid sequence comprising residues 340–347, which they dubbed the UFM1-interacting motif (UFIM)^25^. To support this interaction, they solved the crystal structure of a UFM1-UFIM complex and provided the first structural insight on how UBA5 binds UFM1^25^. Unexpectedly, in this structure the peptide interacts unequally with two molecules of UFM1. Molecule A provides the peptide interaction site and molecule B stabilizes the complex of molecule A with the peptide, but this interaction is not physiologically relevant. A few months later, we determined the crystal structure of the UBA5-UFM1 complex and provided the first structural insight into how UBA5 interacts with UFM1 in the context of the adenylation domain^26^. In that structure UBA5 is a dimer and each adenylation domain is in contact with a UFM1 molecule, thereby generating a complex of two UFM1 molecules with two UBA5 molecules. Similar to the Habisov’s structure, UBA5 binds UFM1 via a region located C-terminal to the adenylation domain that unexpectedly brings UFM1 to the adenylation domain of the adjacent UBA5 molecule. However, in contrast to Habisov’s structure, we found that His 336, which is not part of the UFIM, enters a negatively charged pocket on UFM1 surface, so could contribute to UFM1 binding^26^. Interestingly, although in the Habisov structure the peptide comprising the UFIM sequence has an N-terminal extension, which includes His 336, this residue was not observed in the structure^25^. This therefore prompted us to revisit the question of what is the region C-terminal to the adenylation domain that is required for UFM1 binding and activation and specifically the role of His 336. In this study using binding and activation assays we found that His 336 is required for UFM1 binding and activation. We also determined the crystal structure of UFM1 fused to UBA5 amino acids 334–346, which we dubbed the UFM1-interacting sequence (UIS). In this structure His 336 interacts with UFM1 Glu 38 & 39 and mutating these residues perturbs binding. Unexpectedly, we found that the interaction between UFM1 molecules mimics the interaction of UFM1 with the UIS. Specifically, His 70 of one UFM1, similar to His 336 of the UIS, interacts with Glu 38 & 39 of the other UFM1. All the results of this study improve our understanding of how UBA5 binds UFM1 and demonstrate the importance of His 336 in UFM1 binding and activation.

## Results

### The UFM1-interacting sequence (UIS) of UBA5 comprises of amino acids 334–346

**Figure 1:**
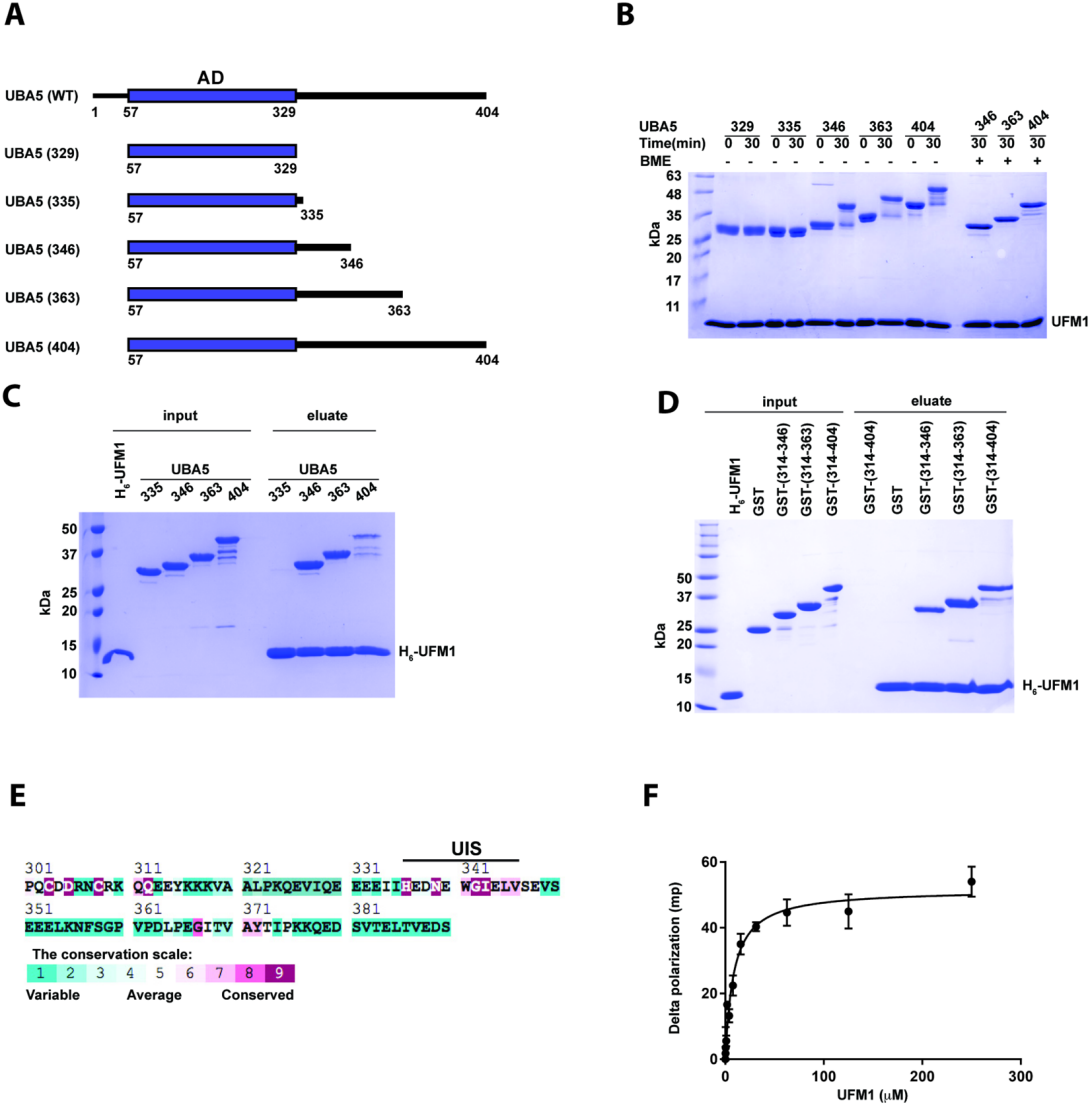
UBA5 possesses a UFM1-binding sequence that is essential for UFM1 activation. (**A**) UBA5 constructs used in this study; AD is adenylation domain. (**B**) Charging assay of UBA5 constructs (10 μM) with UFM1 (50 μM). All constructs start at amino acid 57 and end as indicated by the number in the figure. (**C**) Pull down assay showing binding of UBA5 constructs (numbered as in B) to immobilized H_6_-UFM1. (**D**) Pull down assay showing binding of GST-UBA5 constructs to immobilized H_6_-UFM1. Experiment was performed as in C. (**E**) ConSeq evolutionary conservation analysis^42^ of UBA5. UBA5 residues located C-terminal to the adenylation domain are colored based on their conservation score. (**F**) Fluorescence polarization experiment showing binding of UFM1 to fluorescein-labeled UIS peptide K_D_ = 8.5 μM ± 2.3. Error bars represent the SEM of each measurement.

To map UBA5 residues that are part of the UIS, we tested activation of UFM1 by UBA5 possessing truncations at the C-terminus. While Xie previously demonstrated that UBA5 that ends at amino acid 363 can activate UFM1^24^, longer truncations have not been tested. To that end we generated a battery of UBA5 truncations (Fig. 1A) and tested their ability to form thioester bonds with the UFM1 C-terminus. As expected and shown in Fig. 1B, the UBA5 construct corresponding to the crystal structure of the UBA5 adenylation domain (AA 57–329; PDB 3H8V)^23^ did not show observable activation of UFM1 under our assay conditions. However, UBA5 constructs ending at residue 346 or beyond activated UFM1 efficiently. This therefore enabled us to suggest that residues located C-terminal to amino acid 346 are not required for activation and possibly not part of the UIS. While UBA5 346 comprises almost all the amino acids proposed by Habisov *et al*. to be required for UFM1 binding^25^, it lacks Ser 347. We therefore tested binding of UFM1 to the above UBA5 truncations. To that end we immobilized His_6_-UFM1 to Ni beads and tested whether we could pull down UBA5 constructs. As shown in Fig. 1C, only constructs that extend beyond the adenylation domain to at least amino acid 346 bind UFM1. This therefore suggests that UBA5 346 satisfies not only activation of UFM1 but also binding. While the above experiments have been done in the context of the adenylation domain, we were also interested in whether UBA5 346 satisfies binding to UFM1 even in the absence of the adenylation domain. To that end we fused fragments of UBA5 located C-terminal to the adenylation domain to GST and tested whether His_6_-UFM1 immobilized on Ni beads can pull down these fragments. As shown in Fig. 1D, we detected binding to UFM1 with UBA5 fragments that start at amino acid 314 and go at least to amino acid 346. Our results therefore support Habisov *et al*.’ s peptide array data, but suggest that Ser 347 is not required for UFM1 binding.

Our pull-down experiments demonstrated that the UFM1-interacting sequence of UBA5 is located within a fragment comprising amino acids 314–346. This fragment comprises amino acids (340–346) that were suggested by Habisov *et al*. to be important for UFM1 binding^25^. However, it also includes His 336 that our structure of the UBA5-UFM1 complex suggested is involved in UFM1 binding^26^. This prompted us to investigate which N-terminal residue of the UIS is needed for UFM1 binding. To answer this question, we determined the conservation score of the amino acids within this region. As shown in Fig. 1E, we found that residues 336–346 are highly conserved while residues 314–335 are not conserved. This motivated us to test whether the conserved sequence satisfies binding to UFM1. We synthesized a fluorescently labeled peptide corresponding to amino acids 334–346 of UBA5 and tested its binding to UFM1. As shown in Fig. 1F and Table 1 fluorescence anisotropy experiments with this peptide at different concentrations of UFM1 yielded a K_D_ of 8.5 μM. This value is similar to the K_D_ value achieved measuring by isothermal titration calorimetry for UFM1 and a UBA5 fragment (333–348)^25^. Interestingly, hydrolysis of ATP by UBA5 at various concentrations of UFM1 yielded a K_1/2_ value similar to our measured K_D_, suggesting that the binding we observed is similar to that required for catalysis^27^.

**Table 1:** K_D_ value and statistics from fit to one site specific binding of UFM1-UIS.

One site — Specific binding	Data Set
**Best-fit values**	
Bmax	51.66
Kd (μM)	8.475
**Std**. **Error**	
Bmax	2.313
Kd (μM)	1.551
**95% Confidence Intervals**	
Bmax	46.96 to 56.36
Kd (μM)	5.324 to 11.63
**Goodness of Fit**	
Degrees of Freedom	34
R square	0.9166
Absolute Sum of Squares	1108
Sy.x	5.708
**Number of points**	
Analyzed	36

### Structural insight into the UFM1-UIS interaction

While our mapping of the UIS N-terminal residue suggested that the UIS starts at amino acid 334, we could not rule out the possibility that it includes extra amino acids, which are not part of the UIS. Support for this possibility arrives from Habisov’s structure where electron density was observed only for amino acids 338–346 and not for 333–337, suggesting the latter are not involved in UFM1 binding. Similarly, the UBA5 peptide array data suggested that the required sequence for UFM1 binding consists of amino acids 340–347^25^. However, on the other hand, our structure of the UBA5-UFM1 complex does provide structural insight on residues 334–337^26^. However, since this structure includes the adenylation domain it is still possible that these residues are involved in UFM1 binding only in the presence of the adenylation domain. These structures therefore leave open the question of what is the structural contribution of UBA5 amino acids 334–337 to the interaction with UFM1.

**Figure 2:**
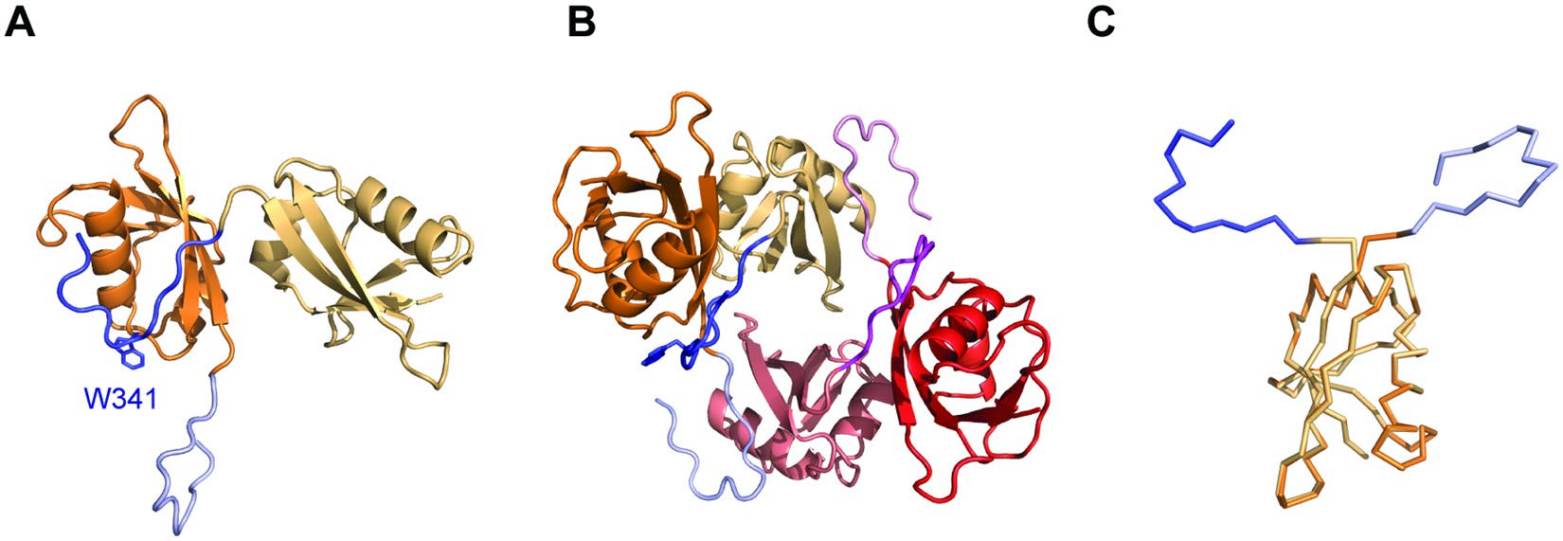
Structure of UIS-UFM1 complex. (**A**) Crystal structure of the UFM1 interacting sequence (UIS) of UBA5 fused to UFM1 (orange). The asymmetric unit contains two molecules A & B. Each molecule possesses a UFM1 part (orange) and a UIS part (blue). (**B**) Two asymmetric units possessing four molecules of UIS-UFM1 form a round structure where each molecule interacts with one molecule via the UFM1 part and another molecule via the UIS part. The two molecules that are colored as in A arrive from one asymmetric unit and the other two molecules come from adjacent asymmetric unit. (**C**) Superposition of the two UIS-UBA5 molecules in the asymmetric unit. Superposition was performed with the UFM1 part of each molecule. Colors are as in A.

**Figure 3:**
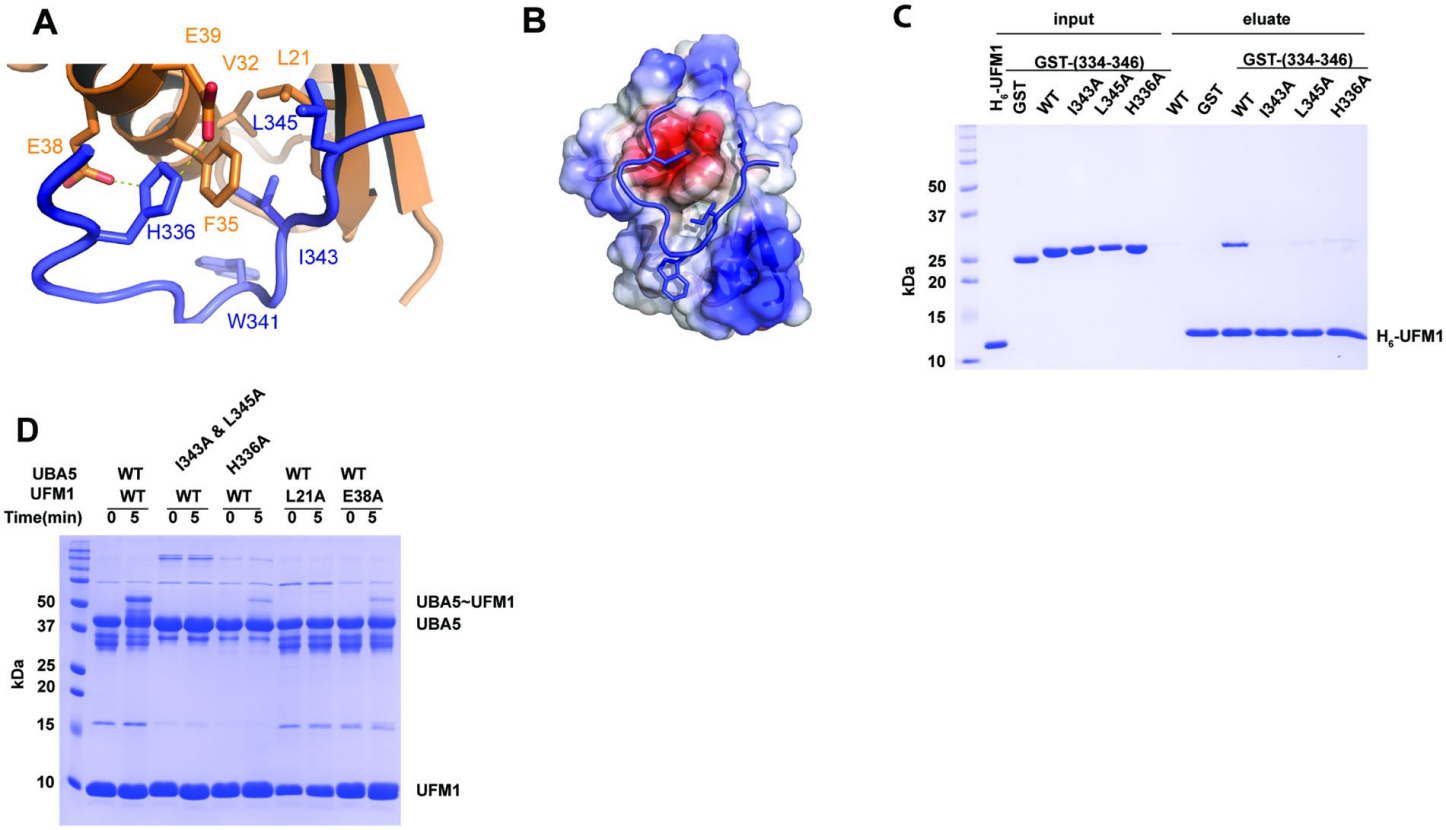
Structural insight into the UIS-UFM1 interaction. (**A**) Contacts between UFM1 (orange) and the UIS of UBA5 (blue). (**B**) Electrostatic surface representation of UFM1 bound to the UIS. (**C**) Pull down assay showing the effect of UIS mutations on binding to UFM1. (**D**) Charging assay of UBA5 WT or mutants (20 μM) with UFM1 WT or mutants (100 μM).

To address this question and with the realization that solving a structure of UFM1 with UIS peptide, similar to Habisov’s structure, could be non-informative for amino acids 334–337, we decided to take a different approach. We fused the UIS to the N-terminus of UFM1 and used this protein to provide structural insight into how the UIS binds UFM1. To that end we determined the crystal structure of that protein at 2 Å resolution (Table 2). Crystals contained two molecules of the UIS-UFM1 fusion in the P4_3_22 asymmetric unit where the UIS from molecule A interacts non-covalently with the UFM1 of molecule B (Fig. 2A). Interestingly, the UIS of molecule B does not interact with the UFM1 of molecule A, but with the UFM1 of a symmetry–related fusion molecule. Two asymmetric units comprising four molecules form a round structure where each molecule binds one molecule via its UIS part and another molecule via its UFM1 part (Fig. 2B). Superposition of the two molecules in the asymmetric unit of the UFM1 part of each fusion shows that the orientation of the UIS relative to the fused UFM1 is different. (Fig. 2C). However, both UISs from each molecule are structurally similar with an RMSD of 0.36 Å, and similarly the UFM1s from each molecule align with an RMSD of 0.18 Å. These data suggest that although one UFM1 binds to the UIS located in the asymmetric unit and the other binds to the UIS that arrives from the symmetry mate, both complexes of UFM1-UIS are similar. The UIS binds to UFM1 on the opposite side of the C-terminal surface of UFM1 and adopts a U-shaped structure with Trp 341 sitting at the base of the U-shape (Fig. 2A). Upon binding to UFM1, the UIS buries 1148 Å^2^ of total surface area. Ile 343 of the UBA5 UIS occupies a hydrophobic cleft on the UFM1 surface composed of Phe 35, Leu 21, Val 23 and Val 32, while Leu 345 binds another hydrophobic patch composed of Leu 21 and Phe 35 of UFM1 (Fig. 3A and B). This part of the structure resembles the Habisov structure (RMSD of 0.5 Å)^25^, suggesting that the interactions in our structure are not affected by the fusion constraints. Both Ile 343 and Leu 345 on the UIS are important for binding UFM1, as mutating either residue to alanine diminishes UFM1 binding (Fig. 3C) and a double mutation severely diminishes UFM1 activation (Fig. 3D). Furthermore, mutating L21A on UFM1 impairs UFM1 activation as well (Fig. 3D).

**Table 2:** Data collection and refinement statistics.

	UFM1-UIS	UFM1
**Data collection**		
Space group	P4_3_22	P2_1_2_1_2_1_
Cell dimensions		
*a*, *b*, *c* (Å)	46.94, 46.94, 201.21	45.28, 56.73, 64.45
α, β, γ°	90.0, 90.0, 90.0	90.0, 90.0, 90.0
Resolution (Å)	2.00 (2.11–2.00)	2.00 (2.11–2.00)
*R*merge	9.6 (83.2)	14.2 (55.3)
Mean*I*/σ*I*	14.7 (2.7)	7.1 (2.7)
Completeness (%)	100.0 (100.0)	99.3 (99.7)
Redundancy	10.1 (10.0)	5.4 (5.7)
**Refinement**		
Resolution (Å)	2.0	2.0
No. reflections	15330	10994
*R*work/*R*free	22.1/23.6	19.8/22.9
No. atoms		
Protein	1395	1161
Ligand/ion	0	0
Water	51	61
B-factor (Å^2^)		
Protein	25.60	15.19
Ligand/ion	—	—
Water	38.47	31.97
R.m.s. deviations		
Bond lengths (Å)	0.010	0.016
Bond angles (o)	1.449	1.749

**Figure 4:**
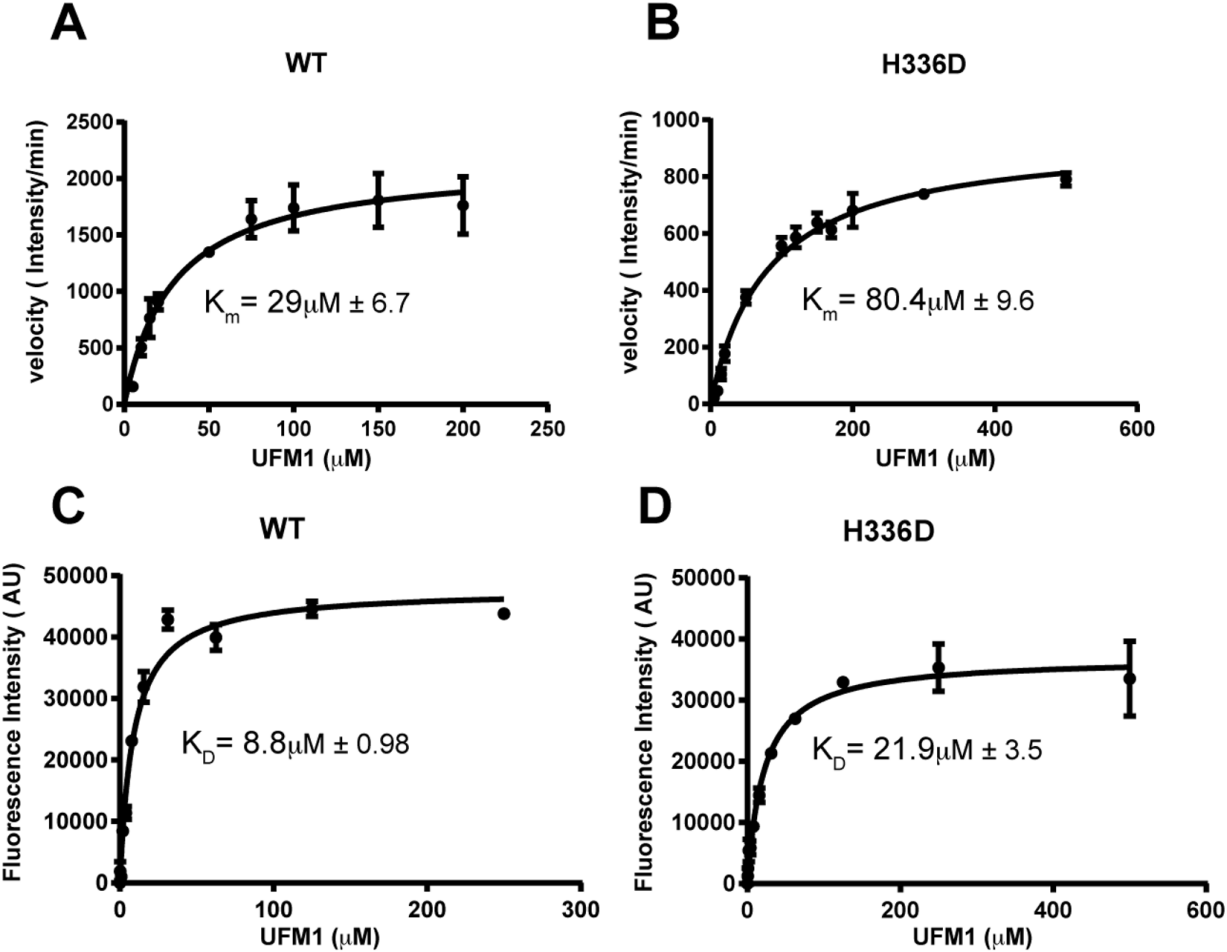
UBA5 His 336 is involved in UFM1 binding and activation. (**A**,**B**) Steady-state kinetic curves of UBA5 (WT or mutant) charging with UFM1. Each rate was measured in triplicates and error bars indicate the SEM. (**C**,**D**) UBA5 Trp fluorescence intensity experiment showing binding of UBA5 WT or mutant to UFM1. Error bars represent the SEM of each measurement.

While the part of the structure described above is similar to Habisov’s structure, in contrast to the latter we also observe amino acids 334–336 in the structure. His 336 binds a negatively charged pocket on UFM1 surface comprised of Glu 38 and Glu 39 and pi-stacks with Phe35 (Fig. 3A and B). This therefore suggests that the contribution of that region is due to the interaction mediated by His 336. Indeed, mutating this histidine to alanine decreases binding to UFM1 as well as activation of UFM1 (Fig. 3C and D). Similarly, mutating UFM1 Glu 38, which forms a salt bridge with UBA5 His 336, to alanine causes an activation defect (Fig. 3D). To further characterize the role of His 336 in UFM1 activation, we compared the steady state kinetics of UFM1 charging by WT UBA5 or UBA5 possessing the H336D mutation. As shown in Fig. 4(A and B) and Table 3, in the presence of the His 336 mutation (H336D), we obtained~three-fold increase in *K*_*m*_ compared to the WT. An increase in the kinetic parameter *K*_*m*_ suggests a decrease in the affinity for substrate. Interestingly, we also found that the above mutation generates a two-fold decrease in the Vmax parameter compared to WT UBA5. Besides the kinetic effect of H336D, we further characterized the effect of this mutation on binding to UFM1. Since UBA5 possesses Trp that is located in the UIS, we measured its fluorescence at increasing concentrations of UFM1, which has no Trp residue in its amino acid sequence. Trp fluorescence is sensitive to changes in its local environment; thereby binding of UFM1 can alter the fluorescence signal. Indeed, as shown in Fig. 4(C and D) and Table 4, in the presence of UFM1 the fluorescence signal of UBA5 WT or H336D increases. In addition, by fitting these intensities to a binding equation (see method section), we can obtain affinity parameters of the UFM1-UBA5 interaction. These binding curves demonstrate that in the presence of the H336D mutation, the *K*_*D*_ increases by~three-fold compare to the WT UBA5 (Fig. 4C and D). Taken together our kinetics and binding experiments suggest that His 336 is required for UBA5 activity as it contributes to the interaction with UFM1.

**Table 3:** Constants and statistics from fits of UBA5 steady-state saturation kinetics to the Michaelis-Menten model.

Michaelis-Menten Analysis	Data Sets	
	UBA5 WT	UBA5 H336D
**Best-fit values**		
Vmax (intensity/min)	2151	943.1
Km (μM)	28.99	80.35
**Std**. **Error**		
Vmax (intensity/min)	151.9	38.08
Km (μM)	6.670	9.656
**95% Confidence Intervals**		
Vmax (intensity/min)	1838 to 2464	865.6 to 1021
Km (μM)	15.26 to 42.73	60.71 to 100.0
**Goodness of Fit**		
Degrees of Freedom	25	33
R square	0.8503	0.9636
Absolute Sum of Squares	1.574e + 006	92611
Sy.x	250.9	52.98
**Constraints**		
Km	Km > 0.0	Km > 0.0
**Number of points**		
Analyzed	27	35

**Table 4:** K_D_ values and statistics from fit to one site specific binding of UFM1-UBA5.

One site — Specific binding	Data Sets	
	UBA5 WT	UBA5 H336D
**Best-fit values**		
Bmax	47820	36944
Kd (μM)	8.853	21.90
**Std. Error**		
Bmax	1306	1511
Kd (μM)	0.9815	3.497
**95% Confidence Intervals**		
Bmax	45165 to 50474	33883 to 40006
Kd (μM)	6.859 to 10.85	14.81 to 28.98
**Goodness of Fit**		
Degrees of Freedom	34	37
R square	0.9712	0.9394
Absolute Sum of Squares	3.443e + 008	4.387e + 008
Sy.x	3182	3444
**Number of points**		
Analyzed	36	39

### The interaction between UFM1 molecules mimics the UFM1-UIS interaction

**Figure 5:**
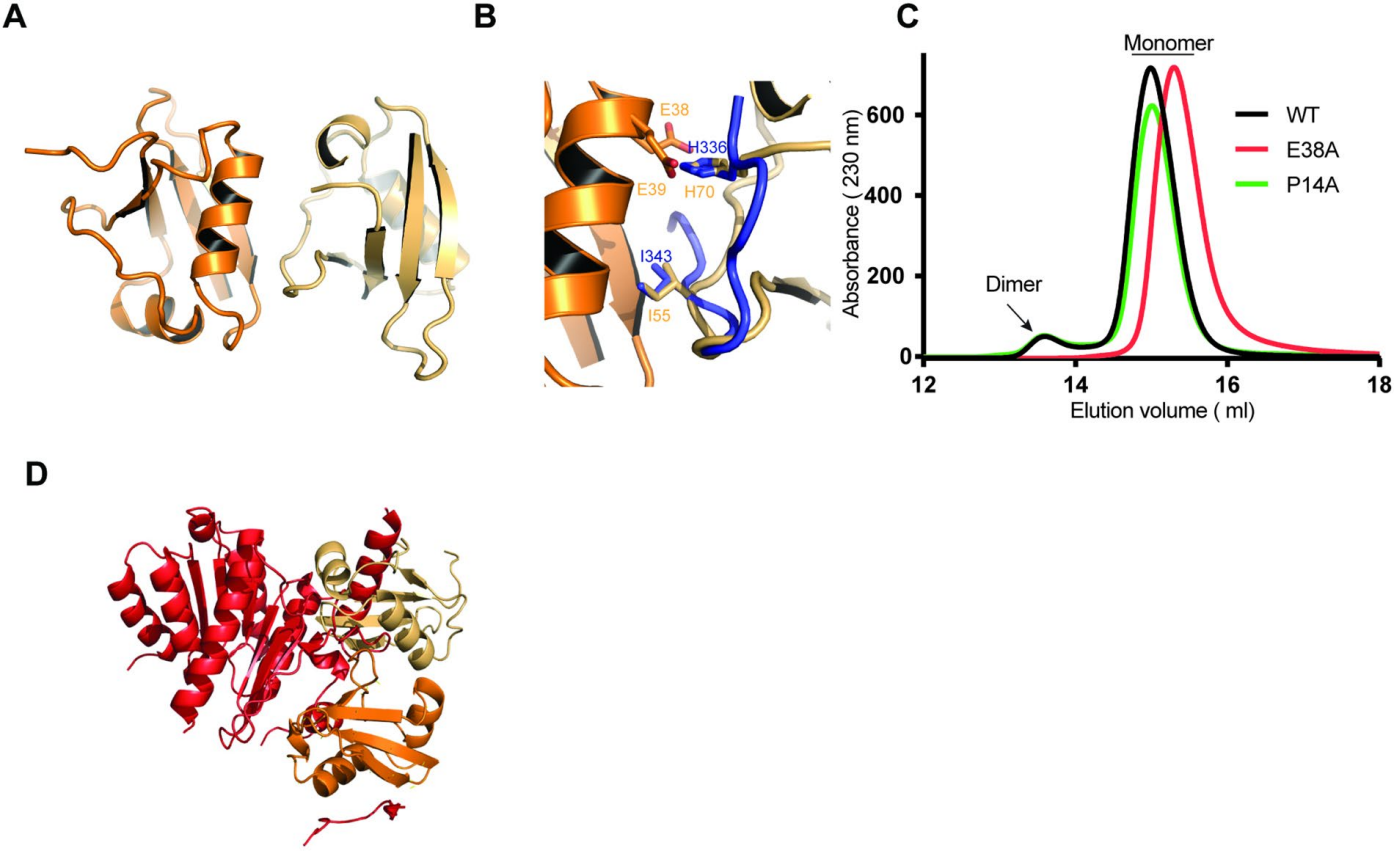
Structural analysis of UFM1 crystal structure. (**A**) Crystal structure of UFM1 showing the beta grasp fold commonly observed in ubiquitin and other UBLs. The asymmetric unit contains two molecules of UFM1. (**B**) Contact between two UFM1 molecules in asymmetric unit mimics the UIS binding to UFM1. Superposition between UFM1-UIS structure and UFM1 alone; the UIS is in blue. The spatial position of UIS H336 and I343 overlap with UFM1 H70 and I55, respectively. (**C**) Gel filtration elution profiles of UFM1 WT or mutants. (**D**) Superposition of UFM1 dimer (orange) with UBA5-UFM1 complex. UBA5 (red) holds the UFM1 dimer in an orientation that generates clashes with the UFM1 molecule that does not interact with the UIS and the adenylation domain, thereby preventing charging of UFM1 dimer.

As the activation of UFM1 by UBA5 is severely impaired in the absence of UIS, suggesting that UIS is indispensable in the mechanism of activation, we asked whether binding of the UIS to UFM1 induces conformational changes in the structure of UFM1 that in turn facilitate UFM1 charging. Therefore to determine the structural changes in UFM1 upon UIS binding, a crystal structure of UFM1 alone is needed. We determined the crystal structure of UFM1 alone at 2 Å resolution (Table 2). The crystals contained two molecules of UFM1 in the P2_1_2_1_2_1_ asymmetric unit with RMSD of 0.14 Å (Fig. 5A). Superposition of this structure with the UIS fused to the UFM1 structure did not show significant conformational changes (RMSD of 0.49 Å). Unexpectedly however, we found that the interaction between the two UFM1 molecules in the asymmetric unit mimics the UIS binding. Specifically, the spatial position of Ile 55 and His 70 of UFM1 overlaps with that of UIS residues Ile 343 and His 336, respectively, which are involved in binding to UFM1 (Fig. 5B and Supplementary Fig. S1). In addition His 70 of one UFM1 molecule, similar to His 336 of the UIS, enters a negatively charged pocket on the surface of the other UFM1 molecule and interacts with Glu 38 & 39 (Fig. 5B). This observation then prompted us to test whether UFM1 forms dimers in solution and whether His 70 is critical for this interaction. Using gel filtration we have found that UFM1 exists not only as monomer but also as dimer (Fig. 5C and Supplementary Fig. S2). However, in the case of the E38A mutation that prevents interaction with His 70, the dimeric form is not seen (Fig. 5C), suggesting that the dimeric interaction is mediated via His 70. Accordingly, UFM1 P14A, which based on the structure is located on the surface of UFM1 and is not involved in dimer formation, does not affect the dimeric form UFM1 (Fig. 5C). Our unexpected observation that a fraction of UFM1 exists as a dimer motivated us to test whether the dimeric form interferes with UFM1 activation. To address this question we superimposed the structure of the UFM1 dimer on our previous structure of the UFM1-UBA5 complex. As shown Fig. 5D, binding of UIS to the UFM1 dimer positions the dimer in an orientation where one of the UFM1 molecules within the dimer clashes with the adenylation domain, thereby preventing activation of UFM1 dimer by UBA5.

## Discussion

While binding of a UBL to the adenylation domain of its cognate E1 is critical for activation, interactions with regions outside the adenylation domain vary between different E1 enzymes^28–34^. In the case of UFM1, Xie has shown that the adenylation domain of UBA5 is not sufficient for UFM1 activation and that a region C-terminal to the adenylation domain is required. Later Habisov *et al*. used binding and structural studies to characterize this region and ended up with a sequence of 8 amino acids (UBA5 340–347) that are needed for UFM1 binding. In this study, we have revisited the question of how UBA5 binds UFM1 and found that His 336, previously not thought to be part of the UIFM1-interacting sequence (UIS), plays a role in UFM1 binding and is required for activation. Therefore we defined that UBA5 possesses a UIS comprising UBA5 residues 334–346.

The effect of UIS mutations was then tested *in vitro* both with binding and activation experiments. However, since the concentrations used in the above *in vitro* assays are probably not equal to the cellular concentrations, it is possible that moderate effect in our *in vitro* assays have a much stronger effect *in vivo* on binding and charging of UFM1. This holds true particularly if the *in vitro* assays were performed with protein concentrations higher than those in the cell. In that case the *in vitro* assay probably underestimates the effect of UIS mutations, since the high protein concentrations compensate for the effect of the mutations. Currently, little is known about the endogenous concentrations of UFM1 and UBA5 in the cell, and the effect of UBA5 mutations in cells is largely evaluated using overexpressed proteins. Furthermore, the limited number of *in vivo* UFMylation assays further challenges the study of the role of the UIS in protein modification by UFM1.

Our results as well as those of Habisov *et al*. suggest that the UIS serves as an anchoring site for UFM1 and thereby facilitates the activation. Our recent work on the complex of UFM1 bound to UBA5 has shown that binding of UBA5 to the UIS assists in bringing UFM1 to the adenylation domain where catalysis takes place. Similar to the structure we present here, in the context of the adenylation domain His 336 interacts with Glu 38&39 of UFM1. This suggests that when UFM1 binds the adenylation domain its interactions with the UIS are reserved. In addition, our observation that the affinity of the UIS to UFM1 is similar to that of the full length UBA5, possessing the adenylation domain, suggests that the latter does not increase the affinity to UFM1 (Figs 1F and 4D). Our observation that the adenylation domain does not contribute to UFM1 binding is not unique to UBA5-UFM1 and holds true for Atg8 and Atg7, where a region outside Atg7 adenylation domain is responsible for Atg8 binding^28, 31, 35^. Currently the events that take place after the binding of UIS to UFM1 and the mechanism of subsequent activation are not clear. We cannot rule out the possibility that the UIS has additional roles besides being an anchoring site for UFM1, and therefore further investigation is required to understand the role of the UIS in UFM1 activation.

Non-covalent interactions that form homo-dimers of UBLs are not common. However, here we unexpectedly found that in solution a fraction of UFM1 exists as dimer. By solving the crystal structure of UFM1 alone and using structure-based mutagenesis, we found that the interactions between two UFM1 molecules mimic how the UIS interacts with UFM1. Specifically, His 336 of the UIS, that here we found to be critical for UIS-UFM1 interaction, is mimicked by UFM1 His 70 that forms similar interaction with UFM1. Currently whether the ability of UFM1 to form dimer is exploited to regulate UFMylation in the cell is not clear and requires further investigation. Interestingly, the UFM1 dimer cannot be activated by UBA5 due to steric clashes, therefore altering the equilibrium between monomer and dimer can regulate the cellular inventory of UFM1 that is potent for activation.

## Materials and Methods

### Cloning and mutagenesis

The human UBA5 and UFM1 open reading frames were synthesized (GenScript) and cloned into pET15b containing an N-terminal HisX6 Tag followed by a TEV protease–cleavage site. UBA5 and UFM1 truncations and mutations were generated by site-directed mutagenesis using the QuikChange mutagenesis kit (Stratagene) following the manufacturer’s protocol. The human UBA5 (57–329) expression plasmid was purchased from Addgene (http://www.addgene.org). UBA5 fragments 314–404, 314–363, 314–346 and 334–346 were cloned into pGEX-4T1. UBA5 (334–346) fused to the N-terminus of UFM1 was generated using PCR and ligation. First, PCR was used to engineer UIS fused to UFM1 at the 5′. Then, this PCR product was cloned into a pET32a vector containing the Trx-His tag followed by a TEV protease–cleavage site.

### Protein expression and purification

The Uba5 constructs were transformed into *E*. *coli* T7 express (New England Biolabs) for expression. The cells were grown in 2xYT medium by inoculating with 1% (v/v) starter culture grown overnight. The culture was grown at 37 °C till the OD600 reached 0.4–0.6 followed by induction with 0.3 mM isopropyl-β-d-thio-galactoside (IPTG). The temperature was shifted to 16 °C after induction and the culture left in the shaker overnight. The cells were harvested by centrifuging at 7000 *g* for 15 mins. The cell pellets were stored at −80 °C till further use. For the UFM1 constructs, the cells were grown with Luria-Bertani (LB) medium and induced with 0.15 mM IPTG.

For the UBA5 constructs with the His tag, the pellets were suspended in a buffer containing 50 mM NaPO_4_ pH  8.0, 500 mM NaCl, 10 mM imidazole, and 5 mM β-mercaptoethanol (BME). Dnase was added to the cell suspension at a final concentration of 1 mM and the cells were homogenized using a homogenizer. Phenyl-methyl sulphonyl fluoride (PMSF) was added at a final concentration of 1 mM, followed by lysing the cells using Microfluidizer (Microfluidics). The cell lysate was centrifuged at 68,900 *g* for 1 h to remove the cell debris. The supernatant was loaded on to the 5 ml His-Trap columns (GE Healthcare). A linear gradient of imidazole (15–300 mM) in 30 column volumes was used to elute the proteins. Fractions containing pure proteins were pooled and dialyzed against the buffer containing 25 mM NaPO_4_ pH  8.0, 300 mM NaCl, and 5 mM β-mercaptoethanol in the presence of TEV protease to remove the His-tag (except UBA5 329 that was directly dialyzed into final buffer). The cleaved protein was loaded onto His-Trap column to separate the His-tag and the protein. The cleaved protein in the flow through was dialyzed into a final buffer containing 20 mM Tris pH 7.5, 50 mM NaCl and 5 mM BME.

The UBA5 fragments fused to GST *viz*, 314–346, 314–363, 314–404, and 334–346 were resuspended in lysis buffer containing 20 mM NaPO_4_ pH  8.0, 150 mM NaCl, and 5 mM β-mercaptoethanol. The cells were homogenized and lysed as described above and the supernatant was loaded on to 5 ml GST-trap columns (GE Healthcare). The bound proteins were eluted using 10 mM L-glutathione. The fractions containing pure proteins were pooled and dialyzed against final buffer 20 mM Tris pH 7.5, 50 mM NaCl and 5 mM BME. The dialyzed proteins were concentrated using ultra centrifugation devices from Amicon, flash-frozen in liquid nitrogen and stored at −80 °C.

The WT UFM1, along with its mutants and the UIS-UFM1 fusion, were purified using His-trap column following the protocol mentioned above for UBA5 constructs except that the buffer composition used for cell suspension was 50 mM NaPO_4_ pH  8.0, 400 mM NaCl, and 10 mM imidazole.

### Crystallization

All crystals were grown using the hanging drop vapor diffusion method at 20 °C, cryo-protected using reservoir solution containing 30% glycerol, and flash-frozen in liquid nitrogen. Crystals of UIS-UFM1 fusion were grown from a 1:1 mix of purified protein (8.1 mg/ml) and well solution containing 0.2 M potassium phosphate di basic and 20% PEG 3350. Crystals appeared in ~5–7 days. Crystals of UFM1 alone were grown from a 1:1 mix of purified UFM1 (7.6 mg/ml) and well solution containing 0.2 M ammonium acetate, 0.1 M sodium citrate tribasic dihydrate pH 5.6 and 30% PEG 4000.

### **Data collection**

The data for the crystals of UFM1-UIS fusion were collected at the MX beamline 14.1 (BESSY-II), Berlin, Germany. The wavelength used for the data collection was 0.9184 Å. The data for UFM1 crystals were collected at the beamline ID30B, ESRF, Grenoble, France. The wavelength used for the data collection was 1.0080 Å. All the data collections were conducted at cryo temperatures. Data sets for both the crystals were processed with iMOSFLM (CCP4)^36^.

### Structure determination and phasing

#### UFM1-UIS fusion

The structure of fusion was solved by molecular replacement using the structure of a mouse hypothetical protein with ubiquitin-like fold (solved by NMR; PDB ID: 1J0G) as the model. The cell content analysis using MATHEWS (CCP4)^37^ suggested two molecules of UFM1-UIS in the asymmetric unit. The molecular replacement was performed using PHASER^38^ by searching for two copies of UFM1 by providing the sequence including the residues from the peptide. The structure solution obtained consists of two monomers of UFM1 in the asymmetric unit with clear density for the residues corresponding to the fused UIS in both monomers. In both the chains, residues corresponding to the peptide are present at the N-terminus [Ser-13 to Val 0 (Chain A) and Ile-11 to Val 0 (in Chain B)], followed by the residues from UFM1. Clear electron density was not found for UFM1 residues 79–83 in either chain. The structure was built using Coot and refined using PHENIX^39^. The final refinement was performed using PDB REDO^40^. The structure was validated for geometry using Molprobity^41^. The structure contains 98.88% of residues falling in the Ramachandran favored region with zero percent outliers.

#### UFM1

The structure of UFM1 was solved using molecular replacement (PHASER). The coordinates from the structure of mouse hypothetical protein (PDB ID:1J0G) mentioned above were used as the model in MR. The MR solution contains two molecules of UFM1 in the asymmetric unit. The structure was built using Coot and refined using PHENIX. The final refinement was done using PDB REDO^40^. The final model of UFM1 is missing residues 1–3 in both chains and 78–83 in chain A and Gly 83 in chain B. The structure was validated for geometry using Molprobity^41^. The structure contains 100% of residues in the Ramachandran favored region.

Coordinates and diffraction amplitudes were deposited in the Protein Data Bank under accession numbers **5IA8** (UFM1-UIS) and **5IA7** (UFM1). Protein–protein interaction surfaces were analyzed using the PISA server at EBI (http://www.pdbe.org/PISA). Figures were generated with PYMOL.

### Pull-down assay

Pull down experiments to detect interactions between UBA5 and UFM1 were done as previously described^26^. Briefly, 6XHis-hUFM1 was immobilized on Ni beads and then binding of UBA5 C-terminal truncations or mutations were tested by analyzing the samples on 15% SDS–PAGE and staining with Coomassie Brilliant Blue.

### Charging assays

UBA5 (WT, mutants or deletions) and UFM1 (WT or mutants) were incubated in reaction buffer consisting of 50 mM Bis-Tris pH 6.5, 100 mM NaCl, 10 MgCl_2_ and 5 mM ATP at 30 °C. Zero time points were taken before ATP was added. Samples were analyzed by non-reducing 15% SDS–PAGE and staining with Coomassie Brilliant Blue.

### Steady-state kinetics assay

Steady-state enzyme kinetic assays were performed at 30 °C in a reaction buffer containing 50 mM Bis-Tris (pH 6.5), 100 mM NaCl, and 10 mM MgCl_2_. UBA5 57–404 WT, or H336D (20 μM) were mixed with increasing concentrations of UFM1 in the presence of 5 mM ATP. The reactions containing UBA5 WT were stopped after 2 min whereas the reactions containing the mutant of UBA5 were stopped after 5 min by the addition of denaturing SDS-PAGE loading dye lacking β-mercaptoethanol and analyzed by SDS-PAGE followed by staining with Coomassie G-250. The product bands corresponding to UBA5-UFM1 were quantified by densitometry with ImageJ software. Reaction velocities were then calculated for each UFM1 concentration and fitted to the Michaelis-Menten equation with GraphPad Prism software.

### UFM1 Gel filtration assay

20 μL of UFM1 WT or mutants at 20 mg/ml were loaded on Superdex 75 10/300 GL (GE Healthcare) pre-equilibrated with 20 mM Tris pH7.5, 50 mM NaCl and 2 mM DTT.

### Fluorescence polarization (FP) assay

UFM1 at various concentrations was incubated with 120 nM of N-terminal fluorescein-labeled UIS peptide in binding buffer (50 mM Hepes buffer pH 7.5, 150 mM NaCl, 5% glycerol and 0.2 mg/ml BSA); with a total of 11 reactions. Then, 30 μl from each reaction were added to the wells of a 384-well microplate and FP was measured at room temperature using a TECAN SPARK 10 M plate reader with excitation and emission wavelengths of 485 nm and 525 nm respectively. The experiment was performed in triplicate. Binding data were analyzed and *K*_D_ value was calculated with the GraphPad Prism software.

### Trp fluorescence intensity assay

UFM1 at eleven different concentrations was incubated with 10 μM of UBA5 57–404 WT, or H336D in buffer 50 mM Bis-Tris (pH 6.5) and100 mM NaCl. Then, 30 μl from each reaction were added to the wells of a 384-well microplate and fluorescence signal was measured at room temperature using a BioTek CYTATION 3 plate reader with excitation and emission wavelengths of 290 nm and 340 nm, respectively. The fluorescence signal of UBA5 alone and UFM1 alone were subtracted from each well possessing UBA5 and UFM1. The experiment was performed in triplicate. Finally intensities were fitted using GraphPad Prism software to binding equation and *K*_D_ values were calculated.

## Electronic supplementary material

Supplementry information
