# Supplementary material for: Novel insights into the interaction of UBA5 with UFM1 via a UFM1-interacting sequence
Source: Sci Rep. 2017 Mar 30;7:508. doi: 10.1038/s41598-017-00610-0 (PMC5428781; doi:10.1038/s41598-017-00610-0)
Supplement: Supplementary file 1 — Supplementry information [file 41598_2017_610_MOESM1_ESM.pdf]

Supplementary information

Novel insights into the interaction of UBA5 with UFM1 via a UFM1-interacting sequence

Prasanth Padala, Walaa Oweis, Bayan Mashahreh, Nadine Soudah, Einav Cohen-Kfir, Emily A. Todd, Christopher E. Berndsen and Reuven Wiener

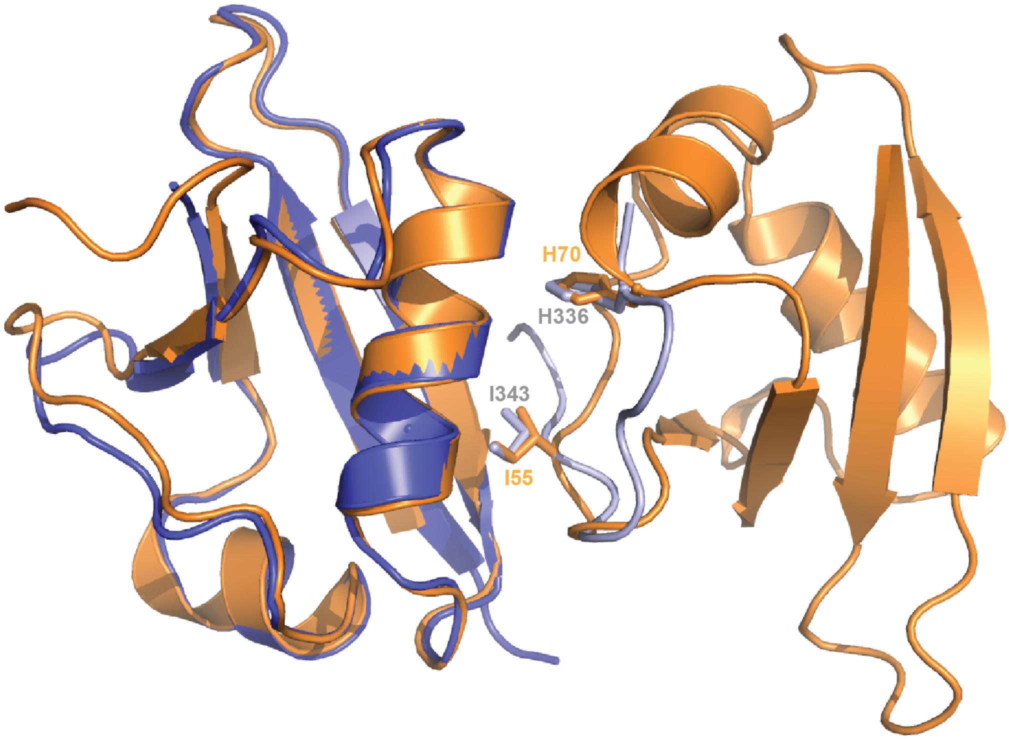

Supplementary Fig. S1. Superposition of UFM1-UIS structure with the structure of UFM1 dimer. The UFM1 molecule (blue) of the UFM1-UIS structure was superimposed on one subunit in the UFM1 dimer (UFM1 dimer is shown in orange). This locates the UIS (light blue) in a position that mimics how the UFM1 molecules in the dimer interact. Specifically, residues H336 and I343 of the UIS overlap with UFM1 residues H70 and I55, respectively.

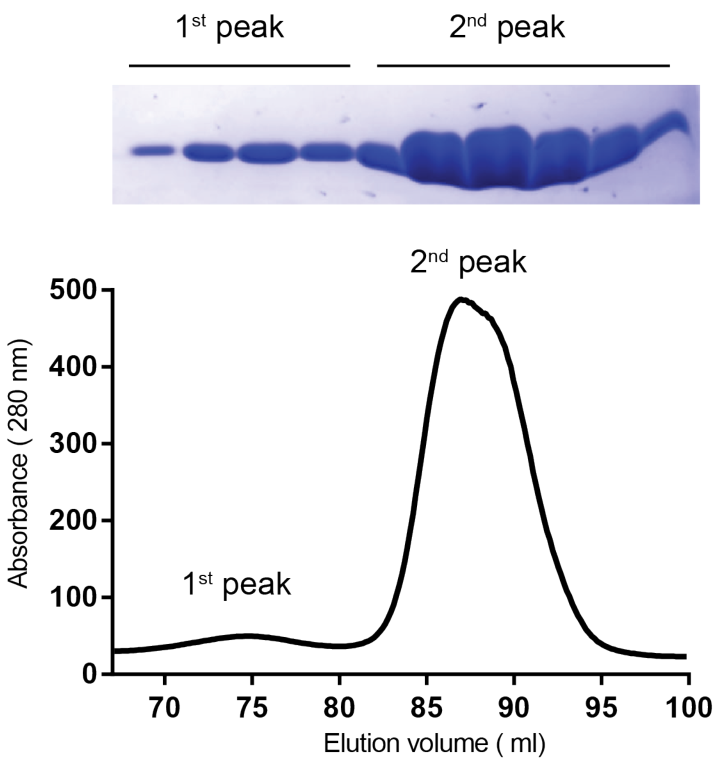

Supplementary Fig. S2. Size exclusion chromatogram and electrophoretic analysis of UFM1. UFM1 purified by Nickel affinity chromatography was concentrated to 22 mg/ml and loaded into a Hiload 16/600 Superdex 75 pg column (GE Healthcare). UFM1 protein eluted from the column was subjected to SDS PAGE (Top panel). Single band for pure UFM1 protein was observed in the fractions corresponding to the dimer (1st peak) and monomer (2nd peak).
